# Answers to Querists

**Published:** 1867-08

**Authors:** 


### CORRESPONDENCE.

ANSWERS TO QUERISTS. *Query* 1*st*.—What is the proper method for obtaining a correct impression of the mouth, for *partial upper* and *lower* sets of teeth? *Answer.*—Some depend upon wax impressions altogether, but as there are few who can obtain a perfectly accurate impression in wax of the mouth, especially where some of the teeth remain, to secure a well fitting plate, plaster of paris is a much more reliable material

Either of the following methods, if carefully observed, will secure as perfect an impression as could be desired. By Professor Austen's method very accurate impressions of all partial cases, as well as of special full cases can be obtained; it is as follows: "Take a wax impression and make a plaster model; in partial cases brush over the teeth of the model one or two layers of thin plaster, to fill up all undercuts, and to make the plate fit loosely; saturate the model with water and mould over it a gutta-percha cup; it should be on the inside from one-fourth to one-half an inch thick, so as to be stiff and unyielding." "The whole inside of the cup must be roughened up with a scaler or excavator in such a way that the plaster can take a firm hold." "In most partial cases the impression must be removed in sections, the inside remaining entire, but the outside and the parts between the teeth coming away separately." In very difficult cases, it is necessary to partially cut through the cup so as to permit its removal in sections with the plaster adherent." "These cups have no handle, but are removed by inserting a plugging instrument into a small hole previously made in the back part of the cup where it is thickest."

Accurate impressions can also be obtained by means of an impression cup for each case, struck up with dies prepared from a wax impression and model.

The following is the method of Dr. Jas. B. Bean, by which he proposes to overcome the tendency of the thin film of plaster, and the frail edges of the impression to break away in taking it from the mouth. "A wax impression is procured from which a set of dies is made and a plate of thin brass swaged so as to approximate a fit, and be easily removed from the mouth." "The edges of this impression cup are trimmed so as to allow the muscles to assume their proper position; a handle is made by soldering a piece of stout brass wire across the plate, from one ridge to the other." "This plate is now warmed over a spirit lamp and coated on the inner surface and edges with gum shellac by rubbing a stick of this material over it while hot." "While the shellac is still in a fused condition, the plate is quickly transferred to a handful of raw cotton, which is wrapped about it and held against the melted shellac until it is cool."

"The superfluous cotton is then removed and the cup is ready for an impression." Very little plaster is necessary, and success is more certain and the impression is more perfect and reliable than with an ordinary impression cup; moreover the impression is very easily removed from the cast by first warming and removing the cup."

*Query 2nd.*—By what means would you prevent rubber from pressing into the joints of the teeth in vulcanite work? *Answer.*—This accident may be prevented by several methods: 1st. By the introduction of plaster of paris, in a dry state, between the blocks, and after well filling the spaces with the plaster in this condition, moistening it with a drop or two of water, and working the thin paste thus formed, into the joints by means of a thin, flat pointed instrument.

This plaster should be allowed to harden before the process of packing in the rubber is commenced. The proper time for the introduction of the plaster is after the piece is invested and all the wax forming the temporary plate is removed ; the plaster being introduced from the palatine or lingual surfaces.

Additional care may be taken, to prevent the rubber from entering the joints, by first introducing the plaster between the blocks from the labial and buccal surfaces ; in the case of partial sets before the piece is placed in the flask ; in the case of entire sets after the model and set are secured in the lower half of the flask. This precaution however, is unnecessary when care is taken to have the plaster, used for investing the piece, mixed very thin. The plaster will accomplish the purpose more effectually, if the spaces between the blocks are ground V shape, the greatest diameter being towards the palatine and lingual surfaces. Such a V shaped space will of course admit of a like shaped mass of plaster, and the pressure exerted upon this by the rubber will tend to wedge it more securely into the joint ; whereas if the blocks are ground in such a manner as to give to the spaces between them parallel walls or sides, no such wedging can take place, and the rubber is much more liable to press into the joint.

2nd. By means of Robert's Os-Artificial. This material answers a good purpose for preventing the rubber from entering the joints. It is mixed very thin, and introduced between the blocks when the grinding of the teeth is completed.

Where this material is used the blocks should be closely ground, no space being left between their approximal surfaces ; and after sufficient time has elapsed for the material to harden from twenty minutes to half an hour being required, the process of investing the piece can be proceeded with. In all cases of vulcanite work it is well to allow the piece to stand from eight to twelve hours after it is invested, before the process of packing in the rubber is commenced. Such delay will allow the plaster to set perfectly, and there will be less danger of the teeth being forced from their proper positions by the pressure necessary to bring the parts of the flask together; this pressure should never be greater than what can be accomplished by the hand alone, without the aid of a lever. To obtain clear joints no little depends upon the quantity of rubber used in packing the piece, and when no more than what is requisite is introduced, hand pressure will, in all cases, prove sufficient to readjust the flask.

---

